# On the flexibility of the multipole model refinement. A DFT benchmark study of the tetra­kis­(μ-acetato)di­aquadicopper model system

**DOI:** 10.1107/S2052252525003355

**Published:** 2025-05-23

**Authors:** Andrej Hlinčík, Tadeáš Fülöp, Peter Herich, Jozef Kožíšek, Karol Lušpai, Lukáš Bučinský

**Affiliations:** a Institute of Physical Chemistry and Chemical Physics, Faculty of Chemical and Food Technology Slovak University of Technology in Bratislava Radlinského 9 SK-812 37 Bratislava Slovak Republic; Universidad de Oviedo, Spain

**Keywords:** Hansen–Coppens model, multipole model, benchmarking, density functional theory, DFT, *ab initio* studies, experimental errors, computational modeling, molecular crystals, materials modeling, molecular simulations

## Abstract

In this study, the flexibility of the multipole Hansen–Coppens (HC) model refinement is investigated on calculated structure factors from a DFT reference for the tetra­kis­(μ-acetato)di­aquadicopper model system (CCDC reference 1811668). The effect of the resolution [sin(θ)/λ], the Cu scattering factor, the inclusion of anisotropic displacement parameters and the positions of the atoms in the refinement are considered in terms of statistical error analysis, residual and deformation density maps, Atoms In Molecules parameters, *d*-orbital populations, and others. The choice of the neutral Cu scattering factor in the HC refinement is found to give the most satisfactory results for most of the monitored parameters, despite the formal Cu^2+^ nature of copper in the studied complex. In order to put the difference between the HC model and the reference DFT (BLYP functional) results on the right scale, several computational chemistry methods (B3LYP, Hartree–Fock, Møller–Plesset perturbation theory and Coupled Clusters Singles and Doubles) were compared with the chosen DFT reference. Differences in the magnitudes of the structure factors and AIM parameters are presented, including considerations of relativistic effects and periodic boundary conditions, *i.e.* a comparison of a molecular crystal calculation versus an isolated molecule in the crystal.

## Abstract 2

The refinement flexibility of the Hansen–Coppens multipole model is tested on DFT calculated structure factors for the tetra­kis­(μ-acetato)di­aquadicopper model system. The Cu scattering factor performs the best of all the options tried for most of the monitored parameters despite the Cu^2+^ nature of the complex studied. The Hansen–Coppens model performs similarly well when comparing deviations among computational chemistry methods.

## 1. Introduction

The X-ray or synchrotron diffraction experiment can be used to determine the crystal structure in a standardized way using the Independent Atom Model (IAM) (Sheldrick, 2015 ▸). In addition to the positions and displacement parameters of the atoms in the crystal structure, it is the Fourier transform of the periodic electron density that defines the structure factors (their moduli squared gives the diffraction intensity). The IAM model itself has two limitations. The first is that the atomic description uses spherically averaged atomic/ionic densities with integer charge, *i.e.* the atomic form factors used are derived for isolated neutral atoms or ions. The second is that the atomic anisotropy/asphericity and bonding effects are missing in the IAM electron density.

It became an obvious goal to go beyond the IAM to extract not only the crystal structure but also the electron density using the measured structure-factor magnitudes. The extension of the IAM was based on atomic orbital (AO) densities, whose parameters were refined against the experimental structure factors (Stewart, 1976 ▸; Hirshfeld, 1977 ▸; Hansen & Coppens, 1978 ▸). The Hansen–Coppens (HC) multipole model became the most widely used approach and was adopted in several programs (Guillot *et al.*, 2001 ▸; Jelsch *et al.*, 2005 ▸; Petříček *et al.*, 2014 ▸; Volkov *et al.*, 2016 ▸; Petříček *et al.*, 2023 ▸):
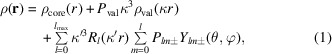
where 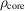 and 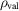 are the core and tabulated valence densities; 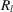 are radial functions used for the aspherical deformation terms; 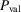 and 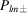 are the refinable populations; 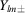 are the density normalized real spherical harmonics; **r**, *r*, 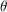, 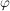 are the position vector, its length and spherical coordinates; and 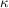 and 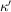 are refinable expansion/contraction parameters.

**Figure 1:**
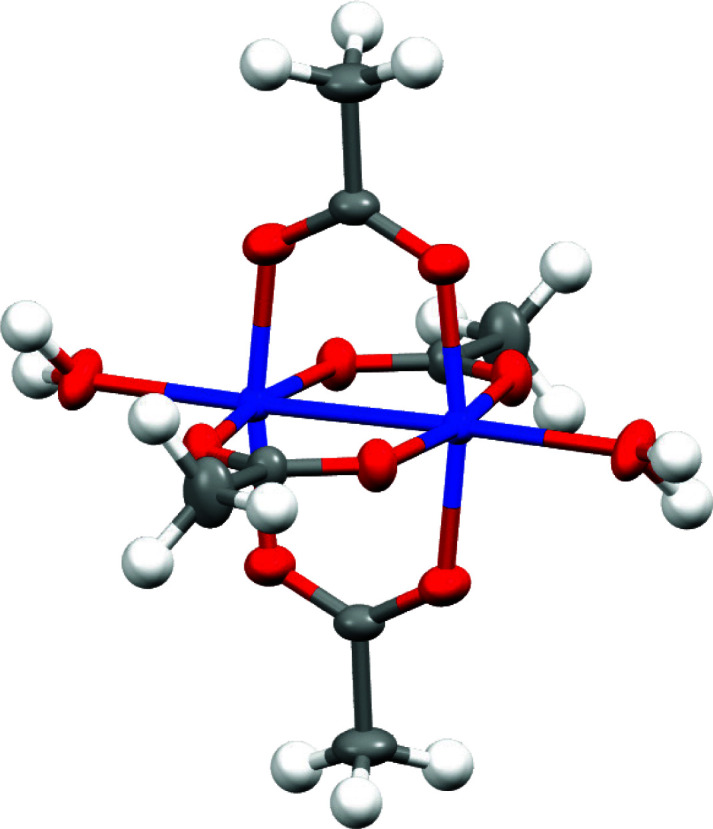
Structure of the tetra­kis­(μ-acetato)di­aquadicopper complex (Herich *et al.*, 2018 ▸): Cu – blue, O – red, C – gray and H – white. All heteroatom ADPs are drawn at a 50% probability level and the hydrogen-atom isotropic radius is 0.3 Å.

Numerous studies have been published on organic compounds, inorganic complexes and periodic materials using the HC model (see below). A considerable number of these studies compare and/or combine theoretical and experimental results. The main focus is a critical evaluation and further improvement of the flexibility of the HC approach, *i.e.* the single-zeta quality of the AO density approach, which became the established HC model (Koritsanszky & Coppens, 2001 ▸). Problems arise due to presence of polarized bonds (C—O, N—O) and atoms with many electrons, such as transition metals, including actinides (Volkov, Abramov *et al.*, 2000 ▸; Volkov, Gatti *et al.*, 2000 ▸; Shishkina *et al.*, 2013 ▸; Zhurov & Pinkerton, 2014 ▸; Vénosová *et al.*, 2020 ▸). In addition, the suitability of the data when a heavy atom is present is another factor to be considered (Stevens & Coppens, 1976 ▸). In particular, there are large differences in the positive curvature along the bond path (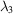) of polarized bonds. This leads to a shift in the bond critical point (BCP) Laplacian value (even a qualitative one, with respect to the sign convention between two heteroatoms), including a quantitative difference in atomic charges, see below. Here, the performance of the HC multipole model refinement is tested on calculated theoretical structure-factor magnitudes. The model system (see Fig. 1 ▸) contains organic ligands with polarized bonds, ligand–metal coordination bonds and a weak metal–metal interaction, providing a valid test case for the aforementioned issues.

Up to now, several approaches have emerged that focus on systematic improvement of the HC model:

(1) The use of static theoretical structure factors in the training of the multipole model prior to the actual experimental structure factors treatment, *e.g.* to pre-determine individual 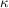 values of the orbital densities in the original model. This approach was among the first used to further tune the HC model quality, referred to as unrestricted multipole model (UMM) and 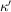 restricted multipole model (KRMM), as initiated by Abramov *et al.* (1999 ▸).

(2) The double-zeta (DZ) basis set quality orbital density representation was another approach to improving HC flexibility (Volkov, Gatti *et al.*, 2000 ▸; Volkov, Abramov *et al.*, 2000 ▸; Volkov *et al.*, 2001 ▸). It has been shown that this improves the topological properties when comparing the DZ-HC electron density with periodic calculations (Volkov & Coppens, 2001 ▸). Verification of the practical applicability of the DZ-HC approach remains a challenge, *i.e.* the data-to-parameter ratio becomes worse, the robustness and stability of the solution is problematic, and structure factors of exceptional accuracy are required (Fischer *et al.*, 2011 ▸).

(3) The Extended Hansen–Coppens (EHC) model (Fischer *et al.*, 2011 ▸; Batke & Eickerling, 2013 ▸, 2016 ▸), which emerged in 2011 with a focus on subatomic resolution (Fischer *et al.*, 2011 ▸), is capable of improving the flexibility of multipole model refinement. It has been shown that at least two multipoles must be used to describe polarizations in the third/fourth (valence) and second/third (core valence) shells of Sc/Y as 3*d*^0^/4*d*^0^ model elements when refining against high-resolution theoretical structure factors (Batke & Eickerling, 2013 ▸). Currently, the EHC model has been used to treat two superconductivity case studies of alpha-boron and MgB_2_ (Fischer *et al.*, 2021 ▸; Langmann *et al.*, 2022 ▸).

(4) The Stockholder Pseudoatoms Approach (SPA) was introduced to avoid fitting in reciprocal space, with adapting the model to a reference density in direct space (a least-squares projection of stockholder atom radial density functions) (Koritsanszky & Volkov, 2004 ▸; Koritsanszky *et al.*, 2012 ▸; Michael & Koritsanszky, 2017 ▸).

However, each of these approaches may suffer from data-to-parameter ratio problems, and the convergence of the least-squares procedure is often difficult to predict without systematic control over the stability of the refinement of all available parameters. Currently, no canonicalization of the HC refinement has taken place, and empirical experience guides the path of parameter constraints in the sequence of steps of an HC refinement.

The development of atomic databases is another example of an HC model analogy. These model databases are designed to be used for protein crystal structure refinement, but can also be well suited for experimentally derived electron density evaluation. Some of these include TAAM (Bąk *et al.*, 2011 ▸; Jha *et al.*, 2020 ▸), ELMAM (Pichon-Pesme *et al.*, 1995 ▸; Zarychta *et al.*, 2007 ▸), ELMAM2 (Domagała & Jelsch, 2008 ▸; Domagała *et al.*, 2012 ▸), the Invariom databases (Dittrich *et al.*, 2004 ▸, 2005 ▸, 2006 ▸, 2013 ▸), the University at Buffalo Pseudoatom Databank (UBDB) (Volkov *et al.*, 2007 ▸; Dominiak *et al.*, 2007 ▸; Jarzembska & Dominiak, 2012 ▸; Kumar *et al.*, 2019 ▸) and last but not least, extremely localized orbitals (ELMOs) libraries (Sironi *et al.*, 2007 ▸; Meyer, Guillot, Ruiz-Lopez & Genoni, 2016 ▸; Meyer, Guillot, Ruiz-Lopez, Jelsch *et al.*, 2016 ▸; Meyer & Genoni, 2018 ▸). The last method (the use of ELMOs) is based on quantum mechanics (QM) reference atomic/fragment orbitals.

A huge step forward in the exploration of crystal and electronic structure is promised by the Hirshfeld Atom Refinement (HAR) method (Jayatilaka & Dittrich, 2008 ▸; Capelli *et al.*, 2014 ▸), labeled NoSpherA2 in the *OLEX2* software (Kleemiss *et al.*, 2021 ▸). Here, the crystal structure and electron density are based on a direct QM method choice and under full control of the QM settings (level of theory/AO basis set *etc*.), now obviously depending on the model Hamiltonian (or DFT method choice) and basis set choice. To emphasize this further, the HC model is a least-squares fit to experimental measurements of hundreds of data points (structure factors) and depends on model flexibility. If the effects of harmonic series truncation and radial flexibility (basis set incompleteness) could be overcome, it would not matter which Hamiltonian or QM method is chosen for the AO density representation in the HC model. Thus, there would be no bias on the information about, *e.g.*, electron correlation and electron density topology. However, as soon as one opens this Pandora’s box, one realizes the thermal smearing, experimental side effects (*e.g.* secondary extinction, absorption *etc*.), resolution limit (the set of experimental structure factors is far from complete) *etc*. that are present. In addition, the HC model fits the electron density directly into the AO density representation, so the one-particle reduced density matrix (1-RDM) is a diagonal entity, and the entire calculation is fast (maximum five minutes for a single CPU in the case of large refinements). On the contrary, a single SCF iteration of a QM method on multiple CPUs may be more time consuming than the five minutes of a rather large HC model job on a single CPU.

To complete this discussion of the different models, the extraction of *N*-representable and idempotent 1-RDMs from the results of a diffraction experiment has a considerable track of references (Genoni & Martín Pendás, 2024 ▸). The Clinton & Massa equations (Clinton & Massa, 1972 ▸) allow the determination of one-electron reduced density matrices from X-ray structure factors (Massa *et al.*, 1985 ▸; Aleksandrov *et al.*, 1989 ▸; Matta & Massa, 2022 ▸; Matta *et al.*, 2022 ▸). The work of Gillet and coworkers uses a combination of X-ray diffraction (direct space) and X-ray Compton scattering (momentum space) data to refine 1-RDMs and shows that such an approach leads to a more robust result compared to using X-ray data alone (Gillet *et al.*, 2001 ▸; Deutsch *et al.*, 2012 ▸, 2014 ▸; De Bruyne & Gillet, 2020 ▸). Another approach to mention is the joint refinement strategy with refinement of the total electron and spin density (unpaired electrons) as an extension of the HC model, which is fully relevant for transition metal complexes or systems with unpaired electrons such as the model compound in this study (Kibalin *et al.*, 2017 ▸; Gueddida *et al.*, 2018 ▸). Besides the HC multipole model approaches mentioned above, another possibility is the Maximum Entropy Method (Sakata & Sato, 1990 ▸; Roversi *et al.*, 1998 ▸; Smaalen & Netzel, 2009 ▸). This method is based on the dynamic electron density evaluated on a lattice and can be classified as model-free (Hamiltonian, basis set). Here, thermal motion is inherently accounted for in the dynamic electron density.

In summary, even standard IAM crystal structure determination uses atomic form factors derived from QM atomic electron densities, and so do the HC-like models when using QM orbital densities in a least-squares fit to experimentally measured structure factors. This initial connection between crystallography and QM is brought to a broader perspective within a single term, namely Quantum Crystallography (Grabowsky *et al.*, 2017 ▸; Genoni *et al.*, 2018 ▸; Genoni & Macchi, 2020 ▸; Macchi, 2022 ▸; Matta *et al.*, 2023 ▸). Here, QM supports the traditional refinements (structure factors, electron density) and crystallography provides the robustness test of the theory behind the structure factors and electron density. The electron density is the property that can be used to extract information from the experimental reference, *e.g.* the electron correlation (in total for HF or as a correction for DFT or other *ab initio* approaches). Thus, the experimental structure-factor magnitudes are of potential use in X-ray Constrained Wavefunction fitting to extract the electron correlation as an inherent part of the experimentally measured evidence (Hupf *et al.*, 2023 ▸; Genoni & Martín Pendás, 2024 ▸). Furthermore, a robust electron density is fully relevant as a reference for quantum computing (Skogh *et al.*, 2024 ▸) or machine learning (ML) predictions (Landeros-Rivera *et al.*, 2022 ▸). Experimentally derived electron densities have been studied in great detail with respect to properties such as superconductivity (Nishibori *et al.*, 2001 ▸; Langmann *et al.*, 2022 ▸) or magnetic properties (Craven *et al.*, 2018 ▸; Thomsen *et al.*, 2019 ▸; Damgaard-Møller *et al.*, 2020 ▸; Gupta *et al.*, 2023 ▸), and not only electron and spin density but also thermal motion (phonons and hydrogen motion) are reported to be extractable from experiment and directly comparable to a QM calculation (Madsen & Hoser, 2014 ▸; Hoser & Madsen, 2016 ▸, 2017 ▸; Destro *et al.*, 2021 ▸; Woińska *et al.*, 2024 ▸).

Here we will focus on coordination complexes and the performance of the HC model (flexibility) with respect to the choice of the actual scattering factor of copper (preferably), since the atomic databanks (Su & Coppens, 1998 ▸; Macchi & Coppens, 2001 ▸) are rich in options. Although a detailed consideration of the transition-metal scattering factor was not the essential goal of previous studies (Yeh *et al.*, 1993 ▸; Farrugia & Senn, 2010 ▸; Wu *et al.*, 2011 ▸; Pinto *et al.*, 2023 ▸), the electron density derived from the diffraction experiment led to correct metal *d*-orbital populations, charges and electron density topology. This amounts to considering *e.g.* single-molecule magnets, metal–metal interactions (Benard *et al.*, 1980 ▸; Hino *et al.*, 1981 ▸; Koritsanszky & Coppens, 2001 ▸; Overgaard *et al.*, 2008 ▸; Hsu *et al.*, 2008 ▸; Tsai *et al.*, 2008 ▸; Farrugia & Senn, 2010 ▸; Wu *et al.*, 2011 ▸; Farrugia *et al.*, 2012 ▸; Bertolotti *et al.*, 2012 ▸; Dinda & Samuelson, 2012 ▸) and agostic interactions (Scherer *et al.*, 2012 ▸). Other aspects of electron density in coordination chemistry compounds (Kucková *et al.*, 2017 ▸) and materials (Schmøkel *et al.*, 2012 ▸; Schmøkel, Bjerg, Larsen *et al.*, 2013 ▸; Schmøkel, Bjerg, Overgaard *et al.*, 2013 ▸; Chimpri & Macchi, 2013 ▸; Schmøkel *et al.*, 2014 ▸), including superheavy elements (Gianopoulos *et al.*, 2019 ▸), have also been considered. The interested reader is referred to detailed reviews (Koritsanszky & Coppens, 2001 ▸; Coppens *et al.*, 2005 ▸; Farrugia & Macchi, 2012 ▸; Jørgensen *et al.*, 2014 ▸; Damgaard-Møller *et al.*, 2020 ▸) on the state of the art of experimentally derived electron density in coordination compounds, highlighting the past and present of various aspects of the field.

The subject of the present study is the tetra­kis(μ-acetato)­diaquadicopper complex, see Fig. 1 ▸, which was recently studied by the HC model and DFT by Bertolotti *et al.* (2012 ▸) and later by Herich *et al.* (2018 ▸) and Malček *et al.* (2020 ▸). It was found that the comparison of the experimental HC model with DFT results leads to a difference in the charge on the coppers of almost 0.8 e in the case of Bertolotti *et al.* (2012 ▸) and 0.4 e when comparing the results of Herich *et al.* (2018 ▸) with those of Malček *et al.* (2020 ▸). In addition, the topology of O—C bonds (BCP Laplacian, 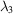 in particular) and/or the charge of heteroatoms such as C1 is another example of a problem with the flexibility of the HC model. Here we will explore the effect of the choice of the central atom scattering factor on the HC refinement results, including the effect of refining (or not) ADPs and positions to show how the convolution of HC model multipoles (Ms) with ADPs affects the refinement. Theoretical data (BLYP structure factors) are used with experimental ADPs (thermal smearing effect) considered (Herich *et al.*, 2018 ▸) unless otherwise stated. Agreement statistics, electron density topology, AIM and *d*-orbital populations will be presented. The results (differences) of the HC model refinement against the theoretical data reference will be compared with the differences in the theoretical (HF, BLYP, B3LYP, MP2, CCSD) results for the structure factors (the reciprocal space), AIM results and *d*-orbital populations (direct space). We will also consider effects such as spin state, periodic boundary conditions, static versus thermally smeared data, and experimental error when commenting on the HC model performance. Theoretical structure factors are available for further testing of HC model flexibility, see the *Data availability* section.

## 2. Computational details

The reference single-point *in vacuo* DFT calculations of the studied system were performed with the *Gaussian16* program package (Frisch *et al.*, 2016 ▸) using the BLYP (Becke, 1988 ▸; Lee *et al.*, 1988 ▸) functional and jorge-DZP-DKH basis set (Canal Neto *et al.*, 2005 ▸; Camiletti *et al.*, 2008 ▸; Jorge *et al.*, 2009 ▸) (Cartesian functions) including scalar relativistic effects via the Douglas–Kroll–Hess second-order Hamiltonian (Douglas & Kroll, 1974 ▸; Jansen & Hess, 1989 ▸), denoted DKH2/BLYP/jorge-DZP-DKH and abbreviated as DKH/BLYP. All open-shell calculations were performed in an unrestricted regime. For comparison, BLYP, B3LYP (Vosko *et al.*, 1980 ▸; Becke, 1988 ▸; Lee *et al.*, 1988 ▸; Becke, 1993 ▸), Hartree–Fock (HF) (Roothaan, 1951 ▸; Pople & Nesbet, 1954 ▸), Møller–Plesset perturbation theory (MP2) (Møller & Plesset, 1934 ▸) and Coupled Clusters Singles and Doubles (CCSD) (Čížek, 1969 ▸; Pople *et al.*, 1978 ▸; Bartlett & Purvis, 1978 ▸; Purvis & Bartlett, 1982 ▸) with the jorge-DZP (Canal Neto *et al.*, 2005 ▸; Camiletti *et al.*, 2008 ▸) basis set were performed with *Gaussian16* for the studied system in the preferred spin state. B3LYP and HF were also calculated with a jorge-DZP-DKH basis set at the DKH2 level of relativistic theory. The Quantum Theory of Atoms in Molecules (QTAIM, abbreviated as AIM) analysis (Bader, 1990 ▸) was performed with the *AIMAll* program package (Keith, 2019 ▸) using the *Gaussian16*-formatted checkpoint (.fchk) file, and the same .fchk file was used as input to *Tonto* (Jayatilaka & Grimwood, 2003 ▸) for the calculation of structure-factor magnitudes. The density = current option was used for the MP2 and CCSD calculations to generate the .fchk files. All calculations were based on the experimental data of Herich *et al.* (2018 ▸), CCDC reference 1811668. The scale factor between the original experimental structure factors and those calculated by *Tonto* was not optimized, and the Stewart method (Stewart, 1976 ▸) was used to describe the thermal motion (atomic displacement parameters, ADPs). *Orca 5.0.3* (Neese, 2012 ▸, 2022 ▸) was used for calculation of Mulliken populations using the DKH/BLYP method. *Crystal17* (Dovesi *et al.*, 2018 ▸) was used for the calculation of static structure factors at the BLYP/pod-DZVP (Peintinger *et al.*, 2013 ▸) level of theory for the preferred spin state.

Owing to the presence of two Cu^2+^ ions with the electronic configuration [Ar]3*d*^9^, the studied complex can have three possible spin states, *i.e.*, the closed-shell singlet (1), the open-shell triplet (3) with parallel spins on each Cu^2+^ and the open-shell broken-symmetry (Noodleman & Norman, 1979 ▸) singlet spin state with opposite spins on each Cu^2+^ (1^BS^). To obtain the open-shell (unrestricted) singlet in the broken-symmetry mode, the triplet-state *Gaussian16* checkpoint file with the guess = (read, mix) keyword was used to restart the unrestricted singlet calculation. The single-point *in vacuo* DKH2/BLYP/jorge-DZP-DKH calculations for the experimental geometry (Herich *et al.*, 2018 ▸) yield the broken-symmetry spin state as the energetically preferred one, which is 6.45 kJ mol^−1^ lower in energy than the triplet, and the singlet is even further away (26.74 kJ mol^−1^), see Table S1 in the supporting information. This is in full agreement with the previous results reported by Malček *et al.* (2020 ▸), therefore the structure factors for the broken-symmetry singlet spin state were used in the multipole model refinement (denoted DKH/BLYP). In addition, the restricted spin state is found to be unstable (keyword stable in *Gaussian16*) (Seeger & Pople, 1977 ▸; Čársky & Hubač, 1991 ▸; Bauernschmitt & Ahlrichs, 1996 ▸). This procedure facilitates a small time-dependent DFT calculation for lowest excited states to prove that the state is a stable ground state or a near excited state (unstable) when a solution from a restricted (closed-shell) type of calculation is inserted into an unrestricted (open-shell singlet) one, see Table S1. The T1 diagnostic (Lee & Taylor, 1989 ▸) within the CCSD calculations in *Gaussian16* showed no significance with values lower than 0.025 for the three spin states of the system studied to evaluate the multideterminant nature in the single determinant representations, see Table S2. Last but not least, CASSCF has also been analyzed (Neese, 2006 ▸; Zein & Neese, 2008 ▸; Duboc *et al.*, 2010 ▸) with 18 electrons in 10 orbitals considered using the jorge-DZP-DKH basis set with SARC/J (Weigend, 2006 ▸; Pantazis *et al.*, 2008 ▸; Pantazis & Neese, 2012 ▸) and AutoAux (Stoychev *et al.*, 2017 ▸) auxiliary basis set activated for the resolution of identity expansion (Neese, 2003 ▸). The CASSCF(18,10) state-specific triplet is a single determinant, and when the singlet is expanded in this triplet solution, it is of the same orbital nature but of a two-determinant character, see Table S3. The two open-shell natural orbitals are each localized on one of the coppers. When the singlet is relaxed in the state-specific CASSCF(18,10) fashion, a two-determinant (|20〉 ± |02〉) closed-shell wavefunction is preferred (see Table S3), with the two natural orbitals shared equally between the two copper atoms, which is still equivalent to the singlet expanded in the triplet solution.

## 3. Refinement strategy

The structure factors computed with *Tonto* (hereafter referred to as ‘computed’) were reduced to the same resolution of sin(θ)/λ, stl ≃ 1.29 Å^−1^, resulting in 12311 original structure factors as experimentally measured by Herich *et al.* (2018 ▸) and taking into account the standard errors from the experiment (except for the single case denoted σ_Gauss_, with a fit to experimental errors including Gaussian noise, see below). The HC model refinements were performed using the *XD2016* program (Volkov *et al.*, 2016 ▸). In all refinements, anomalous dispersion and extinction were turned off, and structure factors with *I*/σ < 3 were rejected from the refinement. The scale factor was optimized in the HC model refinement. We distinguish between refinements where only multipole parameters (Ms) were refined and refinements where ADPs or atomic positions (XYZs) were also refined. Three different resolutions were used: stl ≃ 1.29 Å^−1^, stl < 0.75 Å^−1^, and stl < 0.50 Å^−1^. Three different copper scattering factors were tested, Cu^2+^ (4*s*^0^ 3*d*^9^), Cu^+^ (4*s*^0^ 3*d*^10^) and Cu (4*s*^1^ 3*d*^10^), heteroatoms were treated with neutral scattering factors (unless otherwise stated), with the same 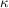 for single element atoms and no 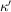 refinement by default. Atomic scattering factors from the BANK SCM databank were used for refinement, as available in the *XD2016* program (Su & Coppens, 1998 ▸; Macchi & Coppens, 2001 ▸). The names of the individual refinement strategies are given in Table 1 ▸, including the cases where the O^−^ scattering factor is used, where different 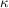 values are used for the atoms of a single element in different chemical environments, or refined (for C and O atoms). For example, the Cu^+^_Ms, ADPs_ labeled refinement uses the scattering factor of Cu^+^ with refinement of multipoles and thermal parameters, while the rest is default. Fig. 1 ▸ was generated using *Mercury 4.0* (Macrae *et al.*, 2020 ▸).

**Figure 2:**
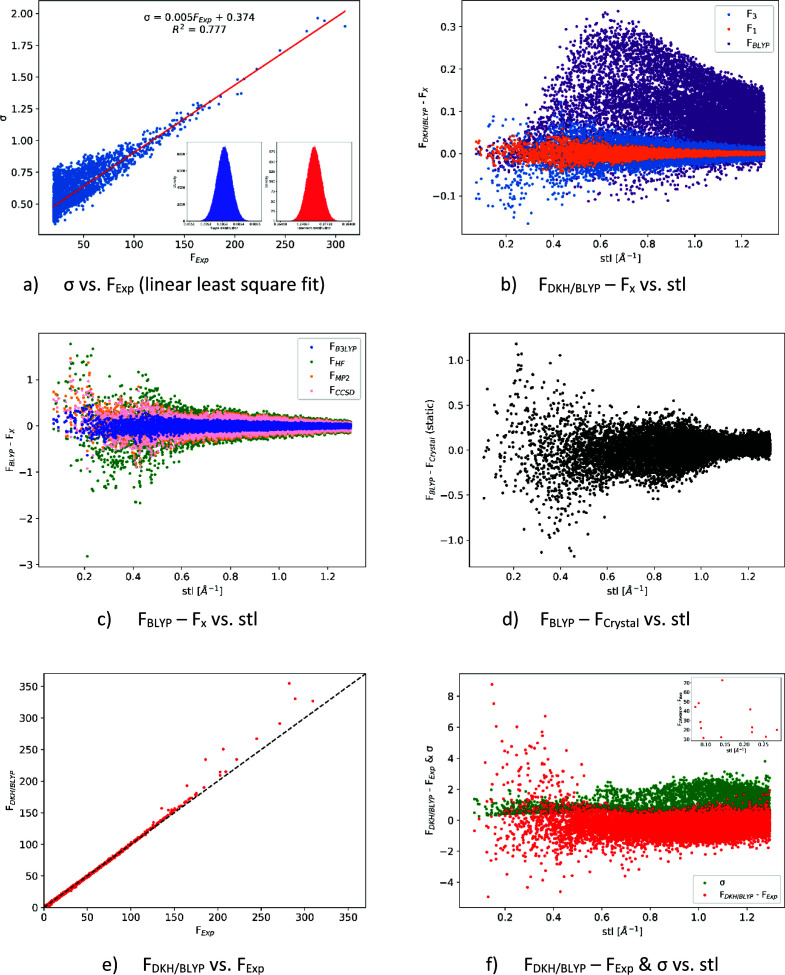
Linear least-squares fit (*a*) of experimental σ as a function of experimental structure-factor magnitude (F_Exp_) and normal distributions of slope (blue) and intercept (red) for σ_Gauss_ data; and different effects in structure-factor magnitudes as a function of (*b*) spin state (*F*_1_ and *F*_3_) and relativistic effects against DKH/BLYP; (*c*) effect of different non-relativistic methods against broken-symmetry BLYP; (*d*) effect of periodic boundary condition on static |*F*| magnitudes; (*e*) correlation of DKH/BLYP and experimental structure-factor magnitudes, black dashed line represents the diagonal; and (*f*) difference of DKH/BLYP calculated and experimental structure-factor magnitudes and experimental errors σ.

The special case of σ_Gauss_ uses smoothed experimental errors that account for Gaussian noise. Here, the error estimate is based on a linear least-squares fit of the experimental errors to the structure-factor magnitudes, assuming the following relationship between 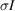, *F* and 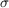:
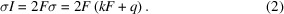


Only structure factors with a magnitude greater than 20 were included in the least-squares fit. The obtained slope (*k* = 0.005312 ± 0.000045) and intercept (*q* = 0.3739 ± 0.0021), see Fig. 2 ▸(*a*), were randomized by introducing the normal distribution (*sk* and *sq*) into the obtained slope and intercept values independently for each individual standard deviation (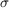) of the structure-factor magnitudes. The intensity error was then calculated using the equation
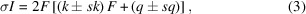
where 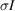 is the standard deviation of the intensity and *F* is the magnitude of the structure factor, *k* is the slope, *sk* is the standard deviation of the slope, *q* is the intercept, and *sq*is the standard deviation of the intercept. To ensure the normal distribution of the slope and intercept, the Python function random.gauss() was used for *sk* and *sq*, see Fig. 2 ▸(*a*).

## 4. Results

### 4.1. The reciprocal-space comparison

Let us first consider the differences shown in Figs. 2 ▸(*b*)–2(*d*), where the structure factors calculated by *Tonto* for different levels of theory are presented. The spin state choice has a very small effect on the structure factors, in the range between −0.15 and 0.15 e. These effects are an order of magnitude smaller than the reported experimental deviation (Herich *et al.*, 2018 ▸). Thus, these effects will be very difficult, if not impossible, to detect in a true measurement. As the DKH/BLYP method was further used in the HC model refinement, the impact of relativistic effects is also addressed in Fig. 2 ▸(*b*). The differences in structure-factor magnitudes due to relativistic effects are larger than the effect of the spin state, *i.e.* in the range of −0.35 to 0.15 e, over the whole stl range. Differences in structure-factor magnitudes for a variety of non-relativistic methods are shown in Fig. 2 ▸(*c*). In this case, some of the differences between methods are at the level of experimental error, at least for the low angular resolution data. The smallest differences with respect to the BLYP reference are found for the B3LYP data, in contrast to the HF, which are largest. Post-HF methods slightly reduce the differences to BLYP. The root-mean-square deviations (RMSDs) between the structure-factor magnitudes are presented in Table 2 ▸ for different ranges of stl using DKH/BLYP or BLYP as reference. The RMSD values for B3LYP, except for the lowest stl range, are lower than the relativistic effects (compared to BLYP). The effect of the periodic boundary condition on the static structure-factor magnitudes (no ADPs included) is shown in Fig. 2 ▸(*d*). This effect is at the same level as the effect of the QM methods (especially HF) and includes the use of a slightly different basis set (pob-DZVP versus jorge-DZP).

The comparison of the experimental structure-factor magnitudes (Herich *et al.*, 2018 ▸) with the DKH/BLYP calculated ones is shown in Fig. 2 ▸(*e*). Some structure factors are significantly larger in the calculated data (they are to the left of the diagonal line), and there is no experimental structure factor with an opposite behavior that is so significant. Fig. 2 ▸(*f*) shows the differences between the calculated and experimental data. It can be seen that the largest differences are for small angular structure factors (stl < 0.50 Å^−1^), and the largest difference is more than 70 e. In all cases of difference (spin state, relativistic effects, QM method choice, experiment) the largest differences are observed for the stl < 0.50 Å^−1^ range, which is the crucial part for a precise description of the bonding and valence electron density in any crystal structure.

### 4.2. The Hansen–Coppens multipole model refinement

The comparison of the *R*^2^ statistics of the HC model refinements against DKH/BLYP is shown in Table 3 ▸. Here we focus on the choice of the Cu scattering factor, the resolution of the structure factors used, and the refinement of the multipoles (Ms) only compared to the ADPs and/or positions (XYZs) included in the refinement. Additional parameters such as the scale factor (between DKH/BLYP and HC model structure-factor magnitudes), the number of independent reflections used (*I* < 3σ) and the ratio of the number of reflections to the HC model parameters accompany the *R*^2^ factors in Table 3 ▸. Table S4 in the supporting information summarizes the same quantities for the additional settings to test the flexibility (and options) of the HC model refinement, as shown in Table 3 ▸. The best *R*^2^ statistic, shown in Table 3 ▸ when only multipoles are refined, is found for the neutral Cu scattering factor (below 0.5%), followed by the Cu^+^ scattering factor *R*^2^ statistic (below 0.7%), with the Cu^2+^ scattering factor performing the worst (*R*^2^ statistic below 1%). Of course, the use of different resolutions plays a role. Interestingly, stl < 0.75 Å^−1^ leads to a worse *R*^2^ statistic for all three scattering factors with only multipoles refined, see Table 3 ▸. The inclusion of ADPs in the refinement naturally lowers the value of *R*^2^ in all cases, most significantly in the case of the Cu^2+^ scattering factor, where it leads to a significant improvement of *R*^2^. In fact, it is not only the statistics that improve: it is shown below that also the AIM volume, charge and *d*-orbital populations of copper in the Cu^2+^_Ms, ADPs_ refinement improve against the DFT reference and are able to be concurrent with Cu_Ms_ and/or outperform the Cu^2+^_Ms_ refinement, see below. This shows the real danger of convolution of HC model parameters (or IAM parameters) with ADPs, where a mathematical minimization strategy leads to improved statistics without full control of physical reasonableness. The comparison of the experimental ADPs with the Cu^2+^_Ms, ADPs_ and Cu^2+^_Ms, ADPs, XYZs_ refinements is summarized in Tables S5 and S6. The inclusion of positions in the refinement reduces the *R*^2^ value by a smaller margin.

**Figure 3:**
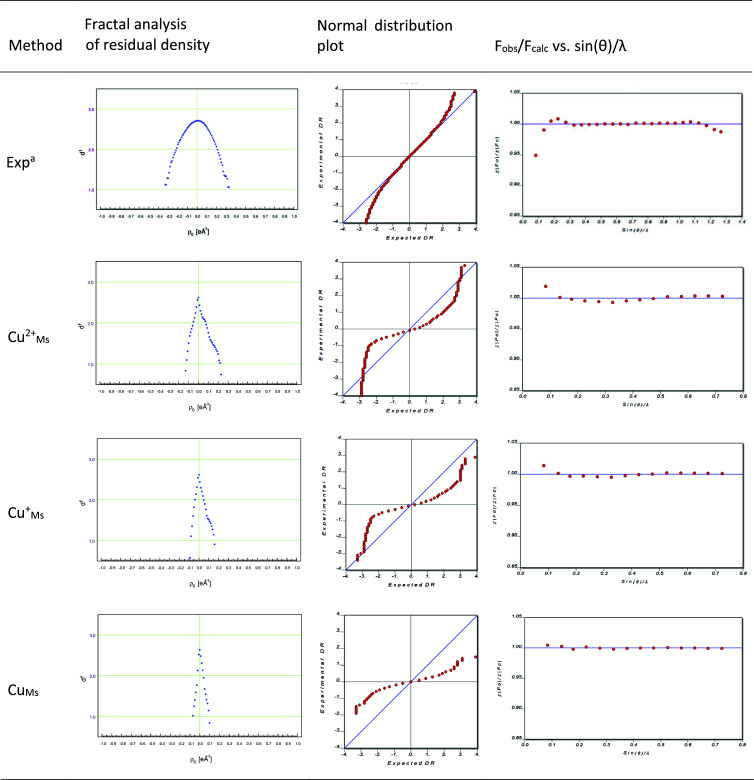
HC model error analysis of theoretically calculated structure factors for different Cu scattering factors and resolution stl < 0.75 Å^−1^. Exp^a^: Herich *et al.* (2018 ▸) used weight *a* = 0.016.

The plots of fractal analysis of the residual density (Meindl & Henn, 2008 ▸), the normal distribution plots (Abrahams & Keve, 1971 ▸; Farrugia, 2012 ▸) and the plots of *F*_obs_/*F*_calc_ versus sin(θ)/λ for selected refinements (Cu-like scattering factor and data resolution) are shown in Figs. 3 ▸ and S1–S3. Considering the effect of the scattering factor on the error analysis of the DKH/BLYP versus the HC model, the most satisfactory results are found for the neutral Cu scattering factor. The neutral Cu scattering factor shows a sharp but non-Gaussian distribution in the fractal analysis (recovering a systematic error between the two approaches used), a normal distribution plot with the lowest slope in the center of the plot, and the *F*_obs_/*F*_calc_ versus sin(θ)/λ plot shows the lowest deviation in the low resolution limit (with a lower deviation throughout the resolution range), see Fig. 3 ▸.

**Figure 4:**
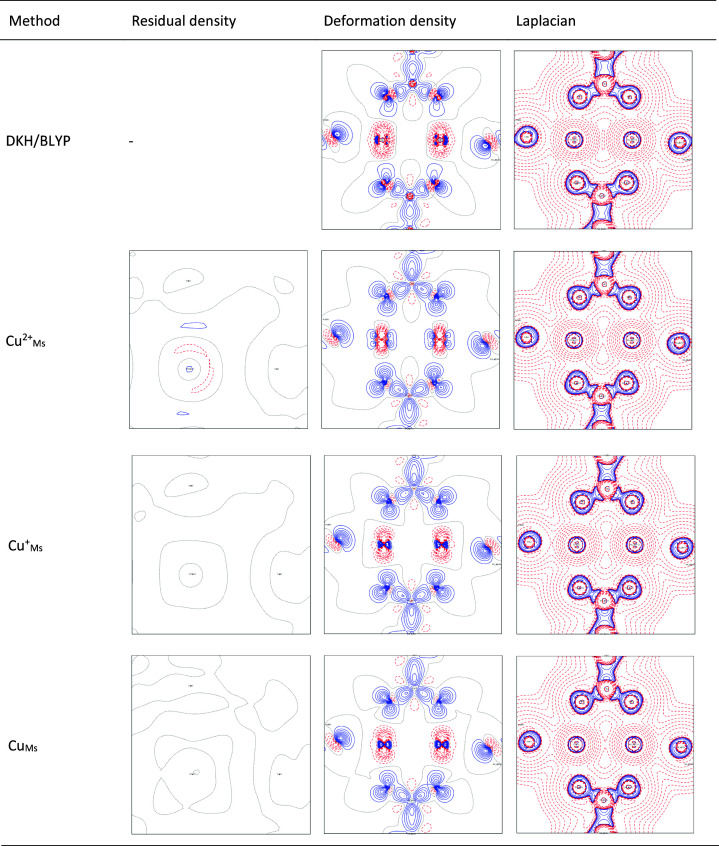
Residual density (atoms Cu1—O1—O3), static electron deformation density (atoms Cu1—O1—O3), contours = ±(0.1, 0.2, 0.3, 0.4, 0.5, 0.6, 0.7, 0.8, 0.9, 1.2, 1.6, 1.9, 2.4) e Å^−3^, and Laplacian (atoms Cu1—O1—O3), contours = ±(0.05, 0.1, 0.2, 0.4, 0.8, 1.6, 3.2, 6.4, 12.8, 25.6, 51.2, 102.4, 204.8, 409.6) e Å^−5^, for theoretically calculated structure factors and for HC model refinement with different Cu scattering factors; resolution stl < 0.75 Å^−1^.

In the case of the normal distribution plots, it is fair to emphasize that experimental standard deviations are used, and therefore the shape of the curve is partially outside the statistics (the slope is not unity, although the weight is zero and the scale factors are refined), as shown in Fig. 3 ▸. Initially, it is noted that a slope different from unity may be an indication of a mis-estimation of the standard deviations (Abrahams & Keve, 1971 ▸). ‘*Thus, a slope of 0.25 would be obtained with data for which the assigned standard deviations were too large, on average, by a factor of 4*’, to quote Abrahams & Keve (1971 ▸). Zhurov & Pinkerton show a theoretical consideration of a normal distribution plot for a double versus single monopole HC model refinement against theoretical data for cronic acid (Zhurov & Pinkerton, 2013 ▸). Their consideration was able to correct the shift of the center of this plot when no scale is applied in the standard double monopole versus single monopole model, but its slope goes to infinity (since the weight was set to one and the standard deviations of the theoretical data were not directly considered) (Zhurov & Pinkerton, 2013 ▸). In the present refinements, when the σ_Gauss_ deviations are made 10 times lower in the Cu^2+^_Ms_ and Cu_Ms_ refinements, the normal distribution plots resemble the steepness of the previous theoretical plots (Zhurov & Pinkerton, 2013 ▸), as shown in Fig. S4. In addition, Cu^2+^_Ms_ shows a shift away from the center of the plot, suggesting a systematic problem in the model, as shown in Fig. S4. In Fig. 4 ▸, the residual density is again the most featureless for the neutral scattering factor, although an isovalue around Cu can be captured. Very similar trends as found for the stl < 0.75 Å^−1^ data in Fig. 3 ▸ are also found for the other resolution sets, as shown in Figs. S1–S3. The same is true for the *R*^2^ statistics of the other HC model variations for Ms and a resolution of stl < 0.75 Å^−1^, as summarized in the supporting information, see Table S4. The use of the O^−^ scattering factor for oxygen atoms helps to improve the *R*^2^ value (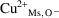 method), as well as the placement of a dummy Cu atom at the same position as the original Cu atom. This allows the original Cu^+^_Ms_ [Ar^18^]3*d*^10^ atom and a dummy Cu_Ms_ 4*s*^1^ atom to be refined in a single refinement. Other options such as refining different kappa (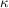) parameters for the same atom type (Cu^2+^_Ms, κ_) and smoothing the structure-factor errors (Cu_Ms_, σ_Gauss_) correlate with the results for their respective scattering factors presented in Table 3 ▸. Another interesting difference is seen in the *F*_obs_/*F*_calc_ versus sin(θ)/λ plot, where the Cu_Ms_ refinement performs best in the low-resolution range. In addition, the results for the HC model fitted to theoretical and experimental data show opposite trends in the low-resolution data range. In the case of the experimental data, the HC model structure-factor magnitudes (*F*_calc_) are larger than the measured data, in contrast to the calculated data. This appears to be the reason for the choice of the Cu^2+^ scattering factor in the HC model refinement against the experimental data (Herich *et al.*, 2018 ▸). However, as will be shown below, the choice of the copper scattering factor does not affect the qualitative interpretation of the results compared to the DKH/BLYP reference, *e.g.d*-orbital populations of copper.

A similar trend as for the *R*^2^ factor (and the refinement statistics plots in Figs. 3 ▸–4 ▸) is seen for the Cu basin AIM volume in Table 3 ▸. It should be noted that the DKH/BLYP Cu basin volume was calculated by extrapolating a polynomial trend line fit to the available *AIMAll* volumes with defined electron density isosurface values, since the package does not provide an electron density isosurface value of 0.001 e Å^−3^. The closest agreement with the reference DKH/BLYP Cu basin volume is found for the Cu_Ms_ refinement, then Cu^+^_Ms_, and the smallest is for Cu^2+^_Ms_, which is actually close to the experimental reference of Herich *et al.* (2018 ▸). The resolution does not play a significant role. The inclusion of ADPs in the refinement only slightly improves the Cu volume with respect to DKH/BLYP, and the Cu-based refinement with ADPs (Cu_Ms, ADPs_) is slightly underestimated with respect to DKH/BLYP. The AIM volumes of Cu in Table S1 are in agreement with the *R*^2^ factor statistics and the results in Table 3 ▸, and are mainly controlled by the choice of the Cu scattering factor.

The deformation density maps of Cu^+^_Ms_ and Cu_Ms_ shown in Fig. 4 ▸ are in better agreement with the DKH/BLYP reference than the Cu^2+^_Ms_ model, probably due to the different reference atomic density. In both the Cu^+^_Ms_ and Cu_Ms_ cases the spherically symmetric 3*d*^10^ atomic population is present, which is apparently also present in the DKH/BLYP promolecular density, while Cu^2+^_Ms_ has a 3*d*^9^ atomic density as a reference. The deformation density around copper is in very good agreement with the 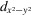 AO density features, which closely resemble the spin (unpaired/open-shell) density when comparing the DKH/BLYP, Cu^+^_Ms_ and Cu_Ms_ deformation densities. In all cases, the deformation density bonding patterns (blue region) are seen around the oxygen and carbon atoms of the acetate groups. However, differences between the HC model results and DKH/BLYP are seen in the C—O bonding region and near the C and O nuclei. In the case of the Laplacian maps visualized in Fig. 4 ▸, small differences can be seen in the middle of the copper and oxygen regions.

### 4.3. Atoms In Molecules and *d*-orbital populations

The AIM charges are shown in Table 4 ▸. The best model, considering only the multipole refinement, is again found for the Cu_Ms_ refinement, which gives the closest value of the Cu charge for all resolutions compared to the DKH/BLYP reference. For the Cu^+^_Ms_ and Cu^2+^_Ms_ scattering factors the Cu charge depends on the resolution: the higher the resolution the more the Cu charge differs from the DKH/BLYP one. It is fair to emphasize that for the low-resolution limit (stl < 0.50 Å^−1^) the Cu charge from the Cu^2+^_Ms_ refinement agrees well with the DKH/BLYP reference. The charges on the other atoms are similar for all refinement methods and in semi-quantitative agreement with the DKH/BLYP reference. The largest deviations from the DKH/BLYP reference are found for the acetate carbons (C1) and oxygen (O5) and the hydrogens of water, see Table 4 ▸. The inclusion of ADPs in the refinement helps to bring the Cu charge closer to the DKH/BLYP value in the case of the Cu^+^_Ms_ and Cu^2+^_Ms_ refinements, but does not improve the charges of the other atoms. In the case of the 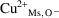 refinement presented in Table S7 of the supporting information, the use of the O^−^ scattering factor helps to improve the oxygen charge, but still does not improve the acetate carbons, and the Cu charge is still the same as for Cu^2+^_Ms_. The best description for the acetate carbons is obtained by the Cu_Ms, κ′_ method, where the 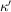 of the oxygens and carbons has been refined. Overall, the difference between the AIM charges of the HC model and the DKH/BLYP results is less than 0.2 e, see Table 4 ▸.

The Cu *d*-orbital populations are examined in Table 5 ▸. The best correlation with the DKH/BLYP reference of *d*-orbital populations only (without considering the population of 4*s* and 4*p* AOs, mentioned in the second footnote to Table 5 ▸) is obtained by the Cu^+^_Ms_ HC model with the two higher-resolution ranges, and Cu^2+^_Ms_ with the 0.50 Å^−1^ resolution. However, the low resolution of 0.50 Å^−1^ leads to the characteristic of having a certain AO-like *d*-orbital population significantly (more than 5%) above two and an underestimation of the 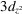 population. There is no constraint on the AO *d*-orbital populations in the HC model, but it seems reasonable to keep them to not more than two, although another fair practice is to give them in percentages when dealing with HC model refinement of experimental data. Cu_Ms_ overestimates the Cu *d*-orbital populations by about 0.90 e, apparently due to the inclusion of the 4*s*^1^ electron density in the refinement, and the AO *d*-orbital populations are well above two, and this looks disappointing for the best-performing scattering factor yet. However, the DKH/BLYP result also includes *s* and *p* AO populations, which together amount to 0.87 e, which can be accounted for using the Cu_Ms_ scattering factor. On the contrary, the other two scattering factors do not take into account the additional population. One can even hypothesize that a subtraction of the average excess of the AO *d*-orbital population over two for all (gross) *d*-orbital populations can lead to corrected net *d*-orbital populations and the 4*s* 4*p* average population of a transition metal for the Cu_Ms_ refinement results.

The inclusion of ADPs in the Cu^2+^_Ms, ADPs_ refinement (for a resolution of stl = 0.75 Å^−1^) leads to larger *d*-orbital populations by about 0.45 e. Thus, the ADP refinement is able to artificially smear errors in the HC model refinement and obtain much closer agreement with the Cu^+^_Ms_ refinement and the DKH/BLYP reference. The inclusion of atomic positions in the refinement with the Cu^2+^ scattering factor does not affect the *d*-orbital populations. It also does not affect the Cu charge and the Cu basin volume. The Cu *d*-orbital populations of special-case refinements (for resolution stl < 0.75 Å^−1^) are presented in the supporting information in Table S8, where the correlation of the *d*-orbital populations and the Cu scattering factor used is shown as for the methods presented in Table 5 ▸.

Nevertheless, it should be emphasized that an electron density HC model is able to distinguish the spin state in some cases (such as a copper center) from the *d*-orbital populations (see also the deformation density maps mentioned above). Here it is found, in full agreement with the DKH/BLYP reference, that 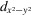 can be considered as an open shell for a properly set local coordinate system (the Cu—Cu bond defines the *z* axis and an acetate oxygen atom the *x* axis).

### 4.4. Clash of *ab initio* and DFT methods

**Figure 5:**
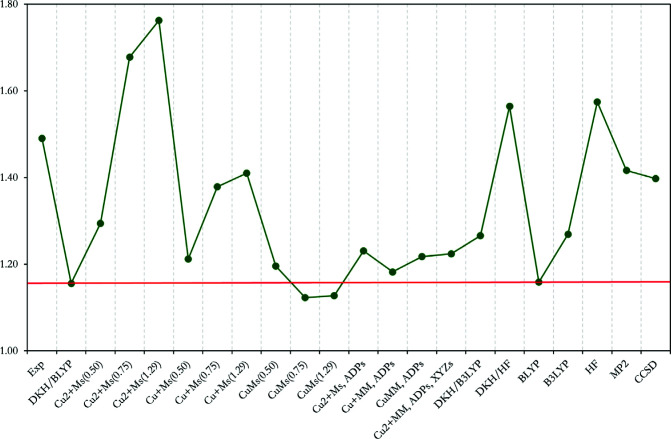
Graphical representation of AIM Cu charge for all previously mentioned HC and QM methods presented in Table 4 ▸ and Table 6 ▸.

To complete the picture, when comparing the accuracy and flexibility of the HC model with a theoretical reference (DKH/BLYP), the performance of the theoretical methods against each other is considered (as already shown for the structure factors). The AIM charges of the different QM methods are presented in Table 6 ▸ together with the experimental results (Herich *et al.*, 2018 ▸), with DKH/BLYP still serving as a reference against which the other methods are compared. A pictorial representation of the AIM Cu charges is shown in Fig. 5 ▸ for all HC models and QM methods, merging the results of Tables 4 and 6. The AIM charges of the remaining heteroatoms are shown in Tables 4 and 6 and in Fig. S5 of the supporting information. The largest differences are found for the Cu AIM charge. In all cases, except for the non-relativistic BLYP calculation, the QM Cu charges are larger compared to BLYP, the largest for HF, which is even higher than the experimental value. The post-HF methods lower the Cu charge by almost 0.20 e compared to HF, which is still almost 0.25 e larger than BLYP, see Table 6 ▸ and Fig. 5 ▸. The differences in the Cu charges closely reflect the differences in the structure factors shown in Fig. 2 ▸(*b*). Relativistic effects have only a small influence on the AIM charges. The AIM charges on other heteroatoms are shifted by less than 0.15 e with respect to BLYP, except for the HF method, which differs by 0.3 e.

Comparing the differences in charges presented in Tables 4 and 6, it can be seen that the theoretical method choice affects the final AIM charge to a similar extent as the HC model fit. We must also add that BLYP is actually the model with the lowest Cu charge among the QM approaches, but this can be considered as an appropriate flexibility check for the HC model.

**Figure 6:**
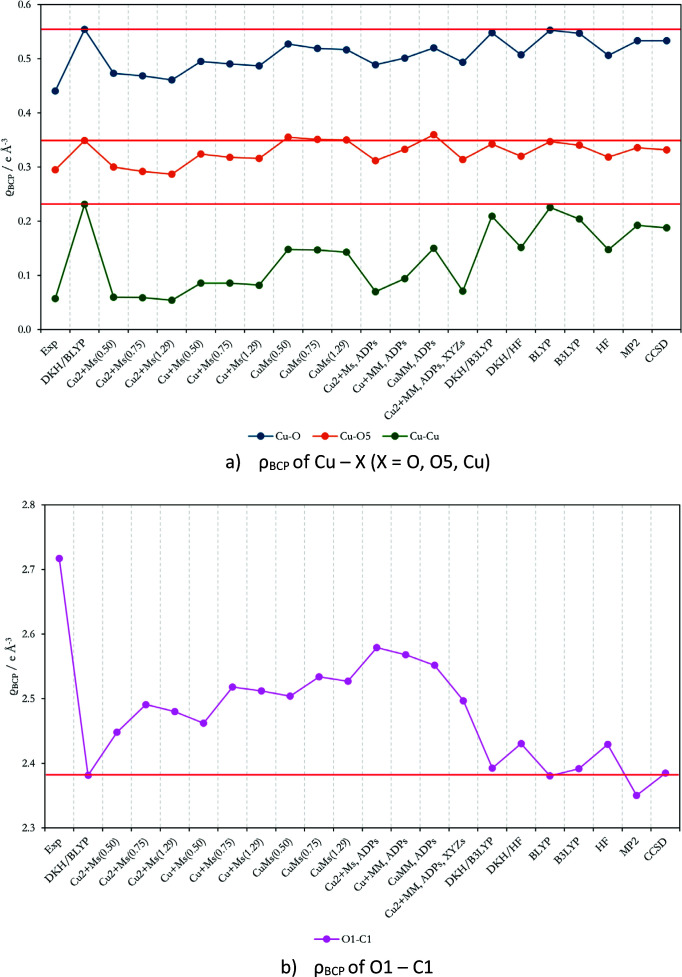
Graphical representation of AIM electron density at bond critical point ρ_BCP_ of chosen interactions; numerical values can be found in the supporting information as an .xlsx file.

Except for AIM charges and atomic basin volumes, the electron density and its Laplacian at bond critical points (BCPs) were also evaluated for all HC refinements and QM approaches, as shown in Figs. 6 ▸ and 7 ▸. The DKH/BLYP BCP electron density and the Laplacian are considered as reference values. The best agreement for the BCP electron density for the Cu—O, Cu—O5 and Cu—Cu bonds is obtained for the Cu_Ms_ refinement (resolution has a very small influence, as do the ADPs and XYZs refinements), see Fig. 6 ▸(*a*). The HC model versus DKH/BLYP agreement is influenced by the choice of the scattering factor (Cu > Cu^+^ > Cu^2+^), see Fig. 6 ▸(*a*). Differences in the QM results copy the trends of the AIM charges, see Fig. 6 ▸. For the O1—C1 covalent bond, the BCP electron density of the HC model refinement results deviates more from the reference DKH/BLYP method when compared to the deviations of the other QM methods, see Fig. 6 ▸(*b*).

**Figure 7:**
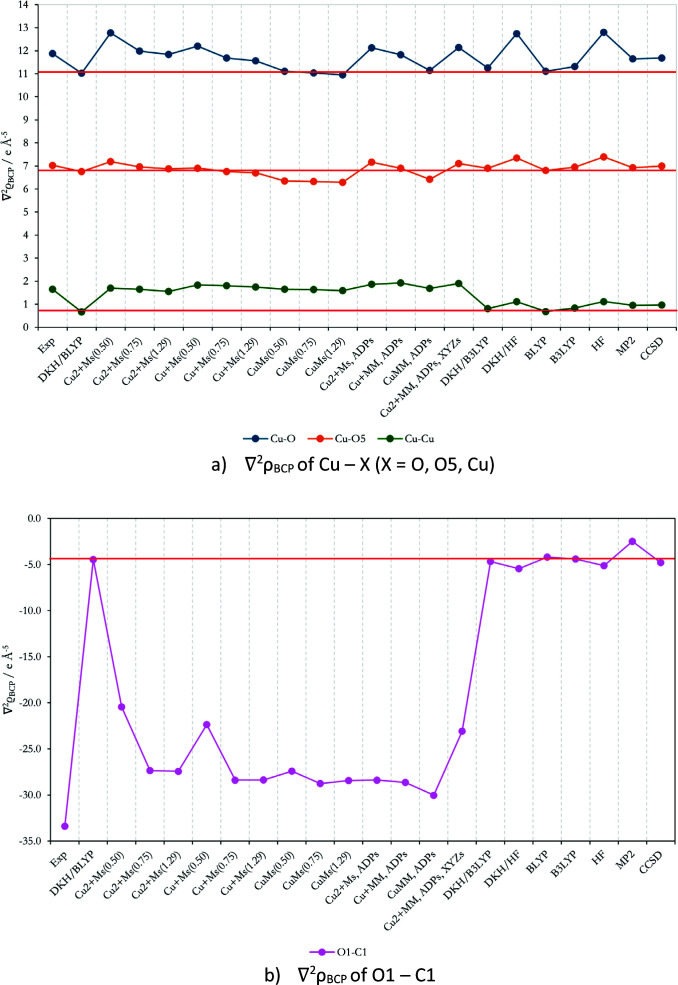
Graphical representation of AIM Laplacian ∇^2^ρ_BCP_ of chosen interactions; numerical values can be found in the supporting information as an .xlsx file.

In the case of the Cu—O, Cu—O5 and Cu—Cu bonds in Fig. 7 ▸(*a*), the BCP Laplacians are largely independent of the HC refinement options. A slightly larger discrepancy for all scattering factors is found for the Cu—Cu bond. The largest and most significant differences are found for the O1—C1 covalent bond, see Fig. 7 ▸(*b*). All QM methods suggest a less negative value of the O1—C1 BCP Laplacian in contrast to the experiment (Herich *et al.*, 2018 ▸) or HC refinement models, which are more negative, further demonstrating the flexibility limits of the HC model in polar bonds even in the presence of a 3*d* transition metal.

## 5. Conclusion

The DKH2/BLYP/jorge-DZP-DKH computational protocol (DKH/BLYP) as a source of reference structure factors has been used to evaluate the performance of the HC multipolar model refinement. The results show that the Cu scattering factor is the best choice to obtain the best agreement for the HC model refinement against the DKH/BLYP reference. The neutral Cu scattering factor performs best in terms of statistics and error distributions, as well as for the AIM charge and basin volume of the Cu atom. The HC model derived *d*-orbital populations for the neutral Cu scattering factor are able to account for the presence of *s*- and *p*-like density in the DKH/BLYP reference. The AIM charges for the remaining atoms are found to be in semi-quantitative agreement with DKH/BLYP, with a deviation of up to |0.20 e|. This deviation is similar to the differences between selected methods of quantum mechanics (BLYP, B3LYP, HF, MP2 or CCSD).

Another important factor present in the experimental data is the thermal motion and the actual refinement of the ADPs. In the case of the most deviated Cu^2+^ scattering factor, the refinement of the ADPs significantly improves the result (partially in *R*^2^) for the AIM Cu charge and Cu *d*-orbital population. This shows the power and struggle of the ADPs: their refinement affects the result, and here is able to improve the electron density quantitatively (showing how much error convolution can hide in ADPs).

Besides focusing on the 3*d* atom description (the choice of the Cu scattering factor) in the HC model, other options were also tested. The use of different 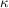 values for C and O atoms in different chemical environments did not improve the HC versus DKH/BLYP atomic AIM charge imbalance and related properties (*e.g.* BCP properties). The use of the O^−^ scattering factor improved only the water oxygen AIM charge. On the contrary, the refinement of 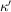 for both C and O improved the C1 charge. Nevertheless, the improvement of the hetero­atomic charges is inconclusive and less readable and interpretable when a 3*d* transition metal is also present. The mixed choice of the Cu^+^ scattering factor (for the 3*d* electron density refinement) with an additional scattering factor for the Cu 4*s* electron density leads to the lowest *R*^2^ value and a well behaved *d*-orbital population for the Cu^+^ moiety, and the 4*s*-like population agrees with a single DKH/BLYP 4*s* density (still missing the fingerprint of the 4*p*-like density in the DKH/BLYP reference).

The spin state can be inferred indirectly from a pure electron density experiment of the compound under study using AO-like *d*-orbital populations as well as by considering the deformation density for the Cu^+^ and Cu scattering factor HC models. However, the differences in the magnitudes of the structure factors between a triplet and a singlet spin state are more than ten times smaller than the experimental errors and are therefore not suitable for distinguishing the spin state from the experimental data.

The accuracy of the experimental data should also be mentioned in terms of resolution, signal-to-noise ratio, anomalous dispersion, absorption and extinction (among others), aside from the flexibility goal of the HC model electron density. The *F*_obs_/*F*_calc_ versus stl plots show that the experiment gives lower structure-factor magnitudes than the HC model for the lowest stl diffractions. When overcoming the current limitations of experiment and electron density description, a least-squares fit of accurate structure-factor magnitudes to a flexible HC model could open new perspectives for quantum crystallography in direct and reciprocal space.

## Supplementary Material

AIM analysis summary. DOI: 10.1107/S2052252525003355/pen5009sup1.xlsx

Structure-factor magnitudes. DOI: 10.1107/S2052252525003355/pen5009sup2.xlsx

Supporting tables and figures. DOI: 10.1107/S2052252525003355/pen5009sup3.pdf

DRYAD data set: https://doi.org/10.5061/dryad.d2547d8bz

## Figures and Tables

**Table 1:** Designation of refinement strategies

Scattering factor		Type of refinement
Cu^2+^	(4*s*^0^ 3*d*^9^)	Ms – multipoles
Cu^+^	(4*s*^0^ 3*d*^10^)	ADPs – anisotropic displacement parameters
Cu	(4*s*^1^ 3*d*^10^)	XYZs – atom positions
		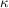 – different 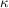 for atoms with similar chemical environment
		O^−^ – scattering factor O^−^ (2*s*^2^ 2*p*^5^) for all oxygens
		σ_Gauss_ – intensity error calculated by least squares and normal distribution
		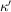 – refinement of 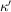

**Table 2:** Root-mean-square deviations (RMSDs) of structure-factor magnitudes relative to the chosen unrestricted singlet 1^BS^ reference (ref) for different ranges of resolution and options/methods 1 and 3 represent restricted singlet and unrestricted triplet DKH/BLYP calculations, respectively.

	ref: BLYP						ref: DKH/BLYP		
Section stl (Å^−1^)	DKH/BLYP	B3LYP	HF	MP2	CCSD	Crystal†	1	3	Exp‡
0.00 to 0.50	0.088	0.133	0.558	0.295	0.286	0.353	0.046	0.017	4.730
0.00 to 0.75	0.136	0.090	0.351	0.201	0.189	0.238	0.033	0.012	2.644
0.00 to 1.29	0.110	0.048	0.176	0.111	0.102	0.141	0.017	0.006	1.292
0.50 to 0.75	0.151	0.064	0.211	0.143	0.129	0.168	0.026	0.010	0.742
0.75 to 1.29	0.103	0.030	0.091	0.075	0.065	0.105	0.011	0.003	0.606

† For static structure factors

‡ Herich *et al.*, 2018 ▸.

**Table 3:** Experimental and DKH/BLYP reference and HC model refinement results Values given include *R*^2^, the number of reflections used in refinements (No. *hkl*), the ratio of the numbers of reflections to HC model parameters (Ratio) and the AIM volume of the Cu basin (*V*_Cu_) with electron density isosurface value of 0.001 e Å^−3^.

Method	stl (Å^−1^)	*R*^2^ (%)	Scale factor	No. *hkl*	Ratio	*V*_Cu_ (Å^−3^)
Exp†	≃ 1.29	3.03	0.9833	339265	1203.1	8.32
						
DKH/BLYP‡	—					9.79§
						
Cu^2+^_Ms_	< 0.50	0.8119	0.9914	677	3.782	8.41
	< 0.75	0.9611	1.0017	2196	12.268	8.40
	≃ 1.29	0.8409	1.0016	9974	55.721	8.30
						
Cu^+^_Ms_	< 0.50	0.6200	0.9935	677	3.782	9.07
	< 0.75	0.6854	0.9982	2196	12.268	9.04
	≃ 1.29	0.6065	0.9985	9974	55.721	9.01
						
Cu_Ms_	< 0.50	0.4451	1.0005	677	3.782	9.76
	< 0.75	0.4728	0.9977	2196	12.268	9.83
	≃ 1.29	0.4597	0.9978	9974	55.721	9.82
						
Cu^2+^_Ms, ADPs_	< 0.75	0.7182	0.9889	2196	8.891	8.65
						
Cu^+^_Ms, ADPs_	< 0.75	0.5763	0.9921	2196	8.891	9.10
						
Cu_Ms, ADPs_	< 0.75	0.4182	1.0012	2196	8.891	9.54
						
Cu^2+^_Ms, ADPs, XYZs_	< 0.75	0.6812	0.9888	2196	7.296	8.63

† Result of Herich *et al.*, 2018 ▸.

‡ DKH2/BLYP/jorge-DZP-DKH single-point calculation.

§ Cubic extrapolation from values reported in *AIMAll* sum file.

**Table 4:** AIM charges from the HC model refinement based on experimental data (Herich *et al.*, 2018 ▸) (Exp), from DKH/BLYP single-point DFT calculations and from HC model refinement based on theoretically calculated structure factors for different Cu scattering factors and refinement settings

Method	stl (Å^−1^)	Cu	O†	O5	C1‡	C2§	H¶
Exp	≃ 1.29	1.49	−1.02	−1.23	1.36	−0.11	0.59
							
DKH/BLYP	—	1.16	−1.12	−1.08	1.55	−0.05	0.56
	< 0.50	1.29	−1.04	−0.96	1.35	0.07	0.49
							
Cu^2+^_Ms_	< 0.75	1.68	−1.04	−0.87	1.41	−0.08	0.44
	≃ 1.29	1.76	−1.03	−0.86	1.38	−0.07	0.43
	< 0.50	1.21	−1.03	−0.93	1.35	0.06	0.48
							
Cu^+^_Ms_	< 0.75	1.38	−1.00	−0.86	1.34	−0.05	0.45
	≃ 1.29	1.41	−0.98	−0.85	1.32	−0.04	0.44
	< 0.50	1.20	−1.06	−0.88	1.39	0.04	0.45
							
Cu_Ms_	< 0.75	1.12	−1.00	−0.86	1.30	0.00	0.45
	≃ 1.29	1.13	−0.99	−0.86	1.28	0.01	0.45
							
Cu^2+^_Ms, ADPs_	< 0.75	1.23	−0.99	−0.92	1.29	0.03	0.48
							
Cu^+^_Ms, ADPs_	< 0.75	1.18	−0.99	−0.90	1.30	0.03	0.47
							
Cu_Ms, ADPs_	< 0.75	1.22	−1.01	−0.86	1.34	0.03	0.44
							
Cu^2+^_Ms, ADPs, XYZs_	< 0.75	1.22	−1.02	−0.95	1.33	0.09	0.49

† Average value of acetate oxygens.

‡ Average value of acetate carbons.

§ Average value of methyl carbons.

¶ Average value of water hydrogens.

**Table 5:** Cu atom *d*-orbital populations from the HC model refinement based on experimental data (Herich *et al.*, 2018 ▸) (Exp), from single-point DKH/BLYP calculations and from HC model refinement based on theoretically calculated structure factors for different Cu scattering factors and refinement settings *d*-tot is the total *d*-orbital population.

Method	stl (Å^−1^)	*z* ^2^	*xz*	*yz*	*x*^2^ − *y*^2^	*xy*	*d*-tot
Exp†	≃ 1.29	2.015	1.909	2.086	1.363	1.985	9.360
							
DKH/BLYP†	—	1.985	1.997	1.994	1.545	1.990	9.510
	< 0.50	1.838	2.151	2.128	1.144	2.282	9.542
							
Cu^2+^_Ms_	< 0.75	1.864	1.972	1.959	1.392	1.982	9.169
	≃ 1.29	1.854	1.910	1.908	1.440	1.971	9.084
	< 0.50	1.971	2.128	2.100	1.342	2.168	9.708
							
Cu^+^_Ms_	< 0.75	1.962	2.042	2.029	1.472	2.042	9.548
	≃ 1.29	1.947	2.005	2.001	1.502	2.061	9.516
	< 0.50	2.159	2.222	2.173	1.631	2.140	10.326
							
Cu_Ms_	< 0.75	2.143	2.207	2.194	1.652	2.204	10.401
	≃ 1.29	2.125	2.182	2.177	1.675	2.238	10.397
							
Cu^2+^_Ms, ADPs_	< 0.75	2.105	2.089	2.077	1.417	1.939	9.627
							
Cu^+^_Ms, ADPs_	< 0.75	2.120	2.111	2.099	1.448	1.972	9.750
							
Cu_Ms, ADPs_	< 0.75	2.217	2.224	2.212	1.561	2.091	10.305
							
Cu^2+^_Ms, ADPs, XYZs_	< 0.75	2.128	2.085	2.080	1.407	1.929	9.629

† Result of Herich *et al.* (2018 ▸).

‡ DKH2/BLYP/jorge-DZP-DKH single-point calculation. 4*s* = 0.469, 4*p*_*z*_ = 0.091, 4*p*_*x*_ = 0.161, 4*p*_*y*_ = 0.150, total(4*s* + 4*p*) = 0.870.

**Table 6:** AIM charges from Herich *et al.* (2018 ▸) HC refinement of experimental data (Exp), from AIM analysis of single-point DFT and from *ab initio* calculations

Method	Cu	O†	O5	C1‡	C2§	H¶
Exp††	1.49	−1.02	−1.23	1.36	−0.11	0.59
DKH/BLYP	1.16	−1.12	−1.08	1.55	−0.05	0.56
DKH/B3LYP	1.27	−1.19	−1.12	1.62	−0.04	0.58
DKH/HF	1.56	−1.39	−1.23	1.85	0.19	0.63
BLYP	1.16	−1.12	−1.07	1.55	−0.02	0.55
B3LYP	1.27	−1.19	−1.11	1.63	−0.01	0.57
HF	1.57	−1.40	−1.22	1.86	0.22	0.62
MP2	1.42	−1.24	−1.14	1.66	0.03	0.59
CCSD	1.40	−1.25	−1.11	1.69	0.09	0.57

† Average value of acetate oxygens.

‡ Average value of acetate carbons.

§ Average value of methyl carbons.

¶ Average value of water hydrogens.

†† Herich *et al.* (2018 ▸).

## Data Availability

The following data are available in the supporting information. Table S1: spin-state preference of DFT and HF methods. Table S2: T1 diagnostics of model systems. Table S3: energy difference between spin states of CAS(18,10) calculations and their determinant composition. Table S4: refinement statistic parameters and AIM atomic basin volume of Cu of special refinement cases. Tables S5 and S6: ADP values of chosen refinements. Figs. S1, S2 and S3: fractal error analysis, normal distribution plots and *F*_obs_/*F*_calc_ versus sin(θ)/λ plots of combination of scattering factors Cu^2+^_Ms_, Cu^+^_Ms_ and Cu_Ms_ with resolutions stl = 0.50, 0.75 and 1.29 Å^−1^. Fig. S4: normal distribution plots of scattering factors Cu^2+^_Ms_ and Cu_Ms_ for refinements with σ_Gauss_ deviation divided by ten. Table S7: AIM charges and Table S8: *d*-orbital populations of special refinement cases. Fig. S5: a graphical representation of AIM charges on O, O5, C1 and H5 atoms for all HC and QM methods. pen5009sup1.xlsx: Excel file with all AIM parameters for all HC and QM methods. pen5009sup2.xlsx: Excel file with *hkl* indices, resolution and all related structure-factor magnitudes and standard deviations for the experimental data. The DRYAD data set offers BLYP, B3LYP, HF and CCSD static and dynamic structure-factor magnitudes with a resolution of up to 6 Å^−1^ and a σ_Gauss_ standard deviation estimate, and is available at https://doi.org/10.5061/dryad.d2547d8bz. Other data are available on request.
