# Supplementary material for: On the flexibility of the multipole model refinement. A DFT benchmark study of the tetra­kis­(μ-acetato)di­aquadicopper model system
Source: IUCrJ. 2025 May 23;12(Pt 4):444–61. doi: 10.1107/S2052252525003355 (PMC12224076; doi:10.1107/S2052252525003355)
Supplement: Supplementary file 3 [file m-12-00444-sup3.pdf]

IUCrJ

Volume 12 (2025)

Supporting information for article:

On the flexibility of the multipole model refinement. A DFT benchmark study of the tetrakis(μ-acetato)diaquadicopper model system

Andrej Hlinčík, Tadeáš Fülöp, Peter Herich, Jozef Kožíšek, Karol Lušpai and Lukáš Bučinský

Table S1 Energy differences of the model system for different spin states and methods

Method	ΔE / kJ mol^{-1}		
	m = 1[a]	m = 1^{BS}	m = 3
DKH/BLYP	26.74[a]	0.00	6.45
DKH/B3LYP	138.67[a]	0.00	2.53
DKH/HF	927.65[a]	0.00	0.28

[a] unstable "wavefunctions", i.e. not an unrestricted ground state

Table S2 T1 diagnostics of the model system

System	State / DFT type	Value
Ne	m = 1 / RKS	0.00519
Ne_2	m = 1 / RKS	0.00520
Anthracene	m = 1 / RKS	0.01073
	m = 1 / RKS[a]	0.02220
dicopper tetrakis(μ-acetato)-diaqua	m = 1 / BS – UKS	0.02257
	m = 3 / UKS	0.02198

[a] CCSD calculation did not converge properly

Table S3 State specific CASSCF(18,10) calculations of the model system for the lowest triplet and singlet roots

Method	m	ΔE / kJ mol^{-1}	Weight	Determinant	CI coefficient
CAS(18,10) m = 3[a]	1	0.00	0.99230	22222222ud	0.70437896
				22222222du	-0.70437896
			0.00475	2222222202	0.06891441
			0.00295	2222222220	-0.05432023
	3	0.20	1.00000	22222222uu	0.99999998
CAS(18,10) m = 1[b]	1	0.00	0.51179	2222222220	0.71539399
			0.48821	2222222202	-0.69872096
	3	0.27	1.00000	22222222uu	0.99999972

[a] Highest natural orbitals, triplet: 101; 92.8 % d(x^2-y^2, Cu_1) and 102; 92.7 % d(x^2-y^2, Cu_2)

[b] Highest natural orbitals, singlet: 101; 46.7 % d(x^2-y^2, Cu1) + 46.7 % d(x^2-y^2, Cu_2) and 102; 46.2 % d(x^2-y^2, Cu_1) + 46.2 % d(x^2-y^2, Cu_2)

Table S4 AIM volume of the Cu basin (V_{Cu}) with electron density iso surface value of 0.001 e.$Å^{-3}$, R factor squared of the HC refinement, scale factor of observed and calculated data from XD2016 package, number of diffractions used in refinements (hkl) and data to (HC model) parameter ratio (ratio) for resolution stl < 0.75 $Å^{-1}$, κ – different kappa for same atom but different surrounding, O^- – scattering factor for O atoms, Cu ($4s^1$) – second copper added to the input and refined by Cu scattering factor, Gauss σ_{Gauss} – structural factor error generated by use of least squares and Gauss distribution, κ' – refinement of κ' for O and C atoms included

Method	V_{Cu} / $Å^{-3}$	R^2	Scale factor	#hkl	ratio
DKH/BLYP	9.79				
$Cu^{2+}_{Ms,\,\kappa}$	8.28	0.9484	1.003	2196	12.133
$Cu^{2+}_{Ms,\,O-}$	8.15	0.8615	1.002	2196	12.268
Cu^{+}_{Ms}, Cu ($4s^1$)	9.78	0.4305	0.996	2196	11.935
Cu_{Ms}, σ_{Gauss}	9.83	0.4948	0.997	2298	12.838
$Cu_{Ms,\,\kappa'}$	9.86	0.4446	0.998	2196	12.200

Table S5 Anisotropic displacement parameters of the model system from (Herich *et al.*, 2018) (Exp), stl < 0.75 Å^{-1} refinements $Cu^{2+}_{Ms,ADPs}$ and $Cu^{2+}_{Ms,ADPs,XYZs}$ of non-hydrogen atoms

Type	Atom						
Exp	Cu	0.006427	0.007692	0.007104	0.000596	0.003294	-0.000012
$Cu^{2+}_{Ms,ADPs}$	Cu	0.005686	0.007155	0.006607	0.000814	0.002925	0.000103
$Cu^{2+}_{Ms,ADPs,XYZs}$	Cu	0.005648	0.007141	0.006601	0.000836	0.002896	0.000124
Exp	O1	0.010946	0.010590	0.016630	-0.001980	0.006430	-0.001919
$Cu^{2+}_{Ms,ADPs}$	O1	0.010730	0.009508	0.016753	-0.002224	0.006438	-0.002014
$Cu^{2+}_{Ms,ADPs,XYZs}$	O1	0.010720	0.009318	0.016792	-0.002040	0.006399	-0.001914
Exp	O2	0.011487	0.009847	0.016160	-0.001800	0.006230	-0.000776
$Cu^{2+}_{Ms,ADPs}$	O2	0.010774	0.009257	0.016113	-0.002266	0.005935	-0.001023
$Cu^{2+}_{Ms,ADPs,XYZs}$	O2	0.010553	0.009290	0.016112	-0.002160	0.005823	-0.001009
Exp	O3	0.010751	0.013900	0.008430	0.002809	0.005118	0.000762
$Cu^{2+}_{Ms,ADPs}$	O3	0.010400	0.014006	0.007405	0.003166	0.004689	0.000923
$Cu^{2+}_{Ms,ADPs,XYZs}$	O3	0.010391	0.013960	0.007347	0.003286	0.004598	0.001100
Exp	O4	0.011983	0.015280	0.008760	0.004123	0.005681	0.001641
$Cu^{2+}_{Ms,ADPs}$	O4	0.011994	0.015285	0.008254	0.004379	0.005952	0.001562
$Cu^{2+}_{Ms,ADPs,XYZs}$	O4	0.011906	0.015250	0.008446	0.004466	0.006083	0.001488
Exp	O5	0.009373	0.015390	0.013410	0.003464	0.001974	-0.003970
$Cu^{2+}_{Ms,ADPs}$	O5	0.008496	0.015310	0.012966	0.003495	0.001558	-0.004201
$Cu^{2+}_{Ms,ADPs,XYZs}$	O5	0.008443	0.015265	0.012854	0.003494	0.001444	-0.004341
Exp	C1	0.011747	0.009343	0.011200	-0.003091	0.006790	-0.002113
$Cu^{2+}_{Ms,ADPs}$	C1	0.011387	0.008911	0.011552	-0.003313	0.006633	-0.002238
$Cu^{2+}_{Ms,ADPs,XYZs}$	C1	0.011735	0.008904	0.011485	-0.003509	0.006861	-0.002311
Exp	C2	0.017410	0.012790	0.018440	-0.006850	0.010070	-0.004380
$Cu^{2+}_{Ms,ADPs}$	C2	0.017181	0.012664	0.018196	-0.006787	0.010005	-0.004332
$Cu^{2+}_{Ms,ADPs,XYZs}$	C2	0.017359	0.012675	0.018214	-0.006869	0.010070	-0.004374
Exp	C3	0.010077	0.011080	0.007680	0.000680	0.004942	-0.000050
$Cu^{2+}_{Ms,ADPs}$	C3	0.010015	0.011250	0.007289	0.000947	0.004824	0.000066

$Cu^{2+}_{Ms,ADPs,XYZs}$	C3	0.010237	0.011170	0.007308	0.000566	0.004978	-0.000134
Exp	C4	0.019950	0.024140	0.009660	0.005890	0.008770	0.001240
$Cu^{2+}_{Ms,ADPs}$	C4	0.019784	0.023934	0.009520	0.006084	0.008769	0.001324
$Cu^{2+}_{Ms,ADPs,XYZs}$	C4	0.019922	0.024034	0.009457	0.006040	0.008794	0.001293

Table S6 Anisotropic displacement parameters of the model system from (Herich *et al.*, 2018) (Exp), stl < 0.75 Å^{-1} refinements $Cu^{2+}_{Ms,ADPs}$ and $Cu^{2+}_{Ms,ADPs,XYZs}$ of hydrogens

Atom	Exp	$Cu^{2+}_{Ms,ADPs}$	$Cu^{2+}_{Ms,ADPs,XYZs}$
H5A	0.036000	0.035993	0.032552
H5B	0.032700	0.033388	0.029666
H2C	0.047700	0.050705	0.051231
H2B	0.055300	0.056935	0.057778
H2A	0.046400	0.049801	0.050678
H4C	0.052000	0.054389	0.055267
H4B	0.067000	0.063038	0.064151
H4A	0.057300	0.059407	0.060272

	stl / Å^{-1}		
	< 0.50	< 0.75	≈ 1.29
Cu^{2+}_{Ms}			
Cu^{+}_{Ms}			
Cu_{Ms}			

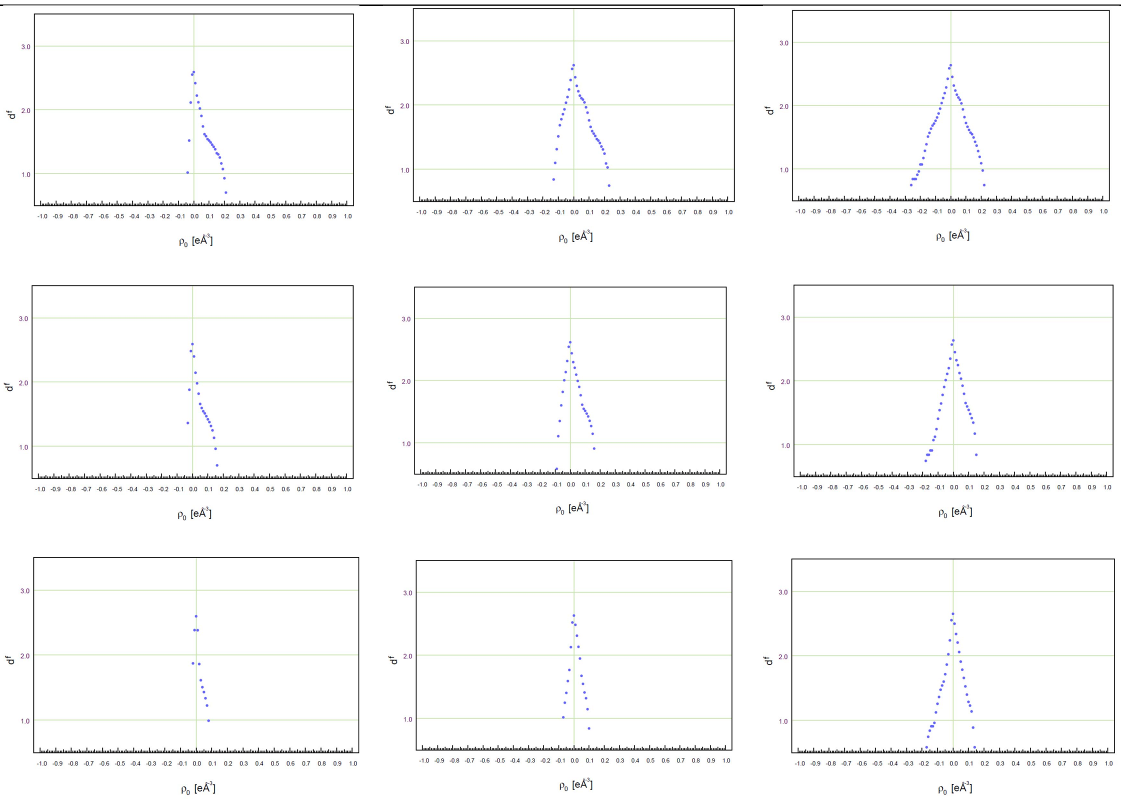

Figure S1 Fractal analysis of residual density for HC refinement of theoretically calculated structure factors (for different Cu scattering factors and resolution)

Method / stl / Å^{-1}	< 0.50	< 0.75	≈ 1.29
Cu^{2+}_{Ms}	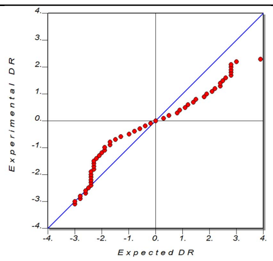	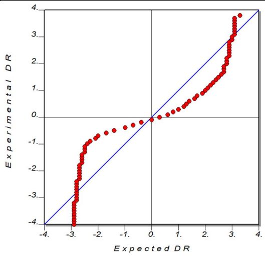	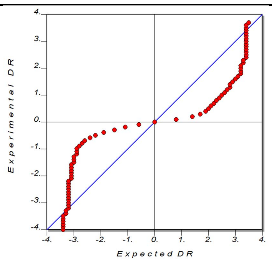
Cu^{+}_{Ms}	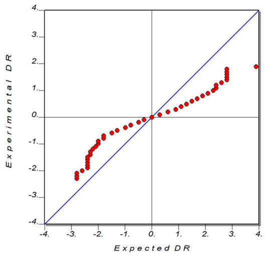	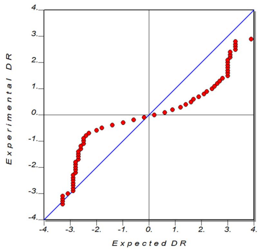	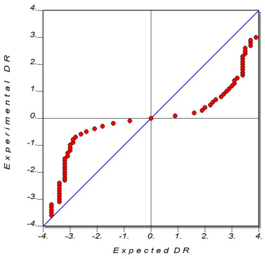
Cu_{Ms}	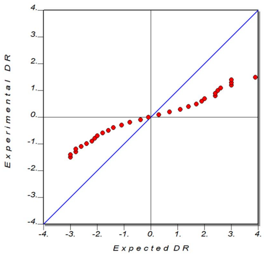	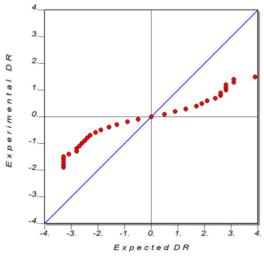	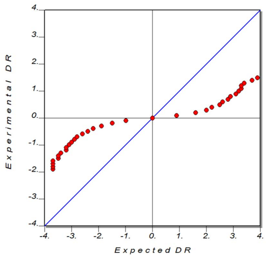

Figure S2 Normal probability distribution plot of theoretically calculated structure factors for different Cu scattering factors and resolution

	stl / Å^{-1}		
	< 0.50	< 0.75	≈ 1.29
Cu^{2+}_{Ms}	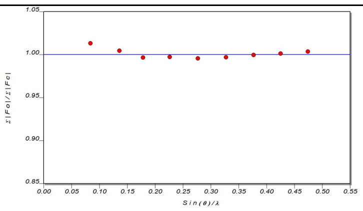	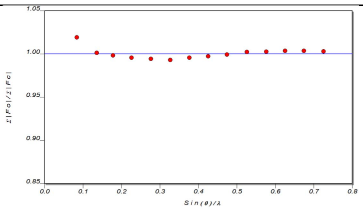	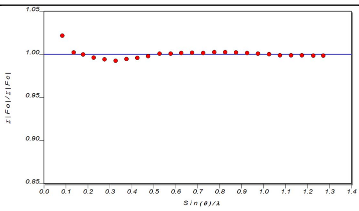
Cu^{+}_{Ms}	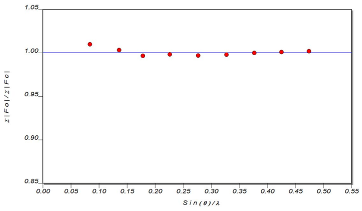	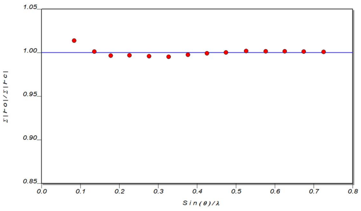	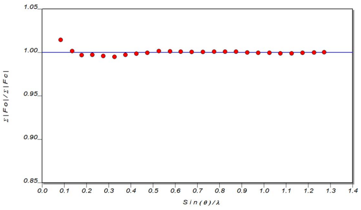
Cu_{Ms}	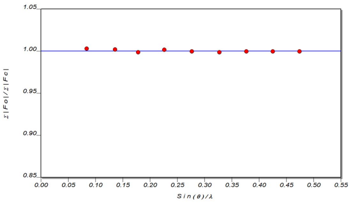	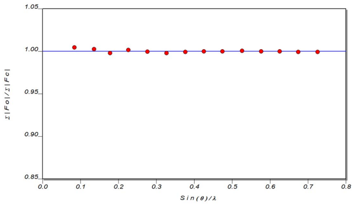	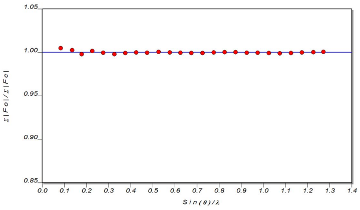

Figure S3 F_{obs}/F_{calc} vs. sin(θ)/λ plot of theoretically calculated structure factors for different Cu scattering factors and resolution

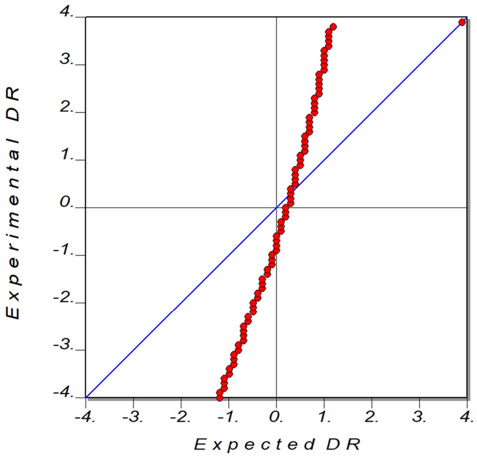

a) Cu^{2+}_{Ms}

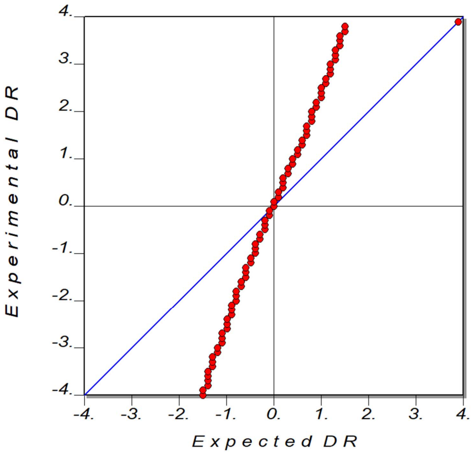

b) Cu_{Ms}

Figure S4 Normal density distribution plots for a) Cu^{2+}_{Ms} and b) Cu_{Ms} refinements with the σ_{Gauss} standard deviations divided by a factor of ten

Table S7 AIM charges from HC refinement (resolution stl < 0.75 Å^{-1}) of theoretically calculated structure factors (for different Cu scattering factors and refinement settings), label as in Table 1

Method	Cu	O[a]	O5	C1[b]	C2[c]	H[d]
DKH/BLYP	1.16	-1.12	-1.08	1.55	-0.05	0.56
Cu^{2+}_{Ms}, κ	1.70	-0.98	-0.86	1.40	-0.31	0.44
Cu^{2+}_{Ms}, O-	1.69	-1.13	-1.08	1.37	0.09	0.50
Cu^{+}_{Ms}, Cu ($4s^1$)	0.92	-1.08	-0.91	1.25	-0.04	0.43
Cu_{Ms}, σ_{Gauss}	1.12	-0.99	-0.87	1.27	0.00	0.47
Cu_{Ms}, κ'	1.11	-1.06	-0.87	1.44	0.03	0.45

average value of: [a] acetate oxygens, [b] acetate carbons, [c] methyl carbons, [d] water hydrogens

Table S8 Cu atom d-populations from HC refinement (resolution stl < 0.75 Å^{-1}) of theoretically calculated structure factors (for different Cu scattering factors and refinement settings), label as in Table 1

Method	z^2	xz	yz	x^2-y^2	xy	d-tot
DKH2/BLYP [a]	1.985	1.997	1.994	1.545	1.990	9.510
Cu^{2+}_{Ms}, κ	1.861	1.966	1.952	1.386	1.977	9.142
Cu^{2+}_{Ms}, O-	1.838	1.925	1.917	1.359	1.932	8.971
Cu^{+}_{Ms}, Cu ($4s^1$)[b]	1.937	2.008	1.996	1.457	2.006	9.404
Cu_{Ms}, σ_{Gauss}	2.129	2.205	2.203	1.650	2.214	10.401
Cu_{Ms}, κ'	2.147	2.212	2.197	1.655	2.212	10.424

[a] 4s = 0.469, $4p_z$ = 0.091, $4p_x$ = 0.161, $4p_y$ = 0.150, tot_{4s+4p} = 0.870, [b] second Cu have evenly distributed-populations of 0.309 (total population 1.546)

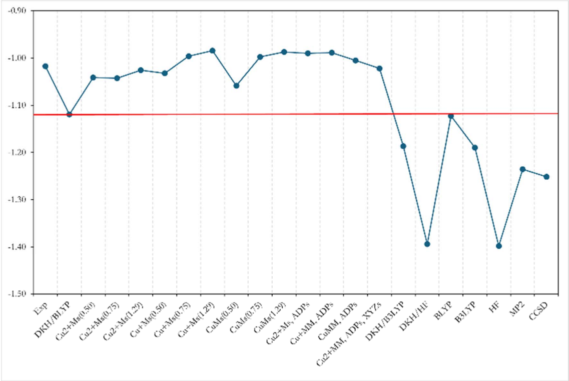

a) O[a]

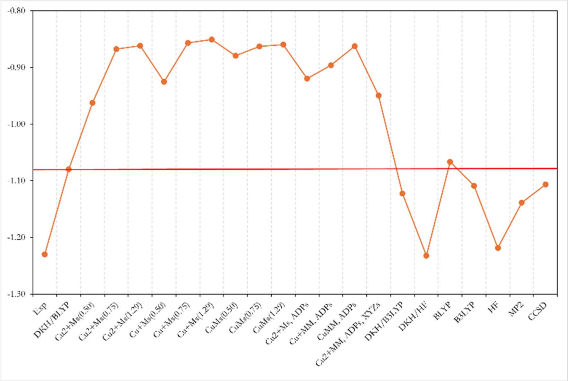

b) O5

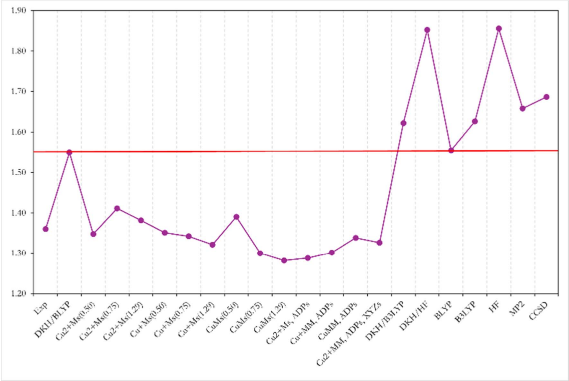

c) C1[b]

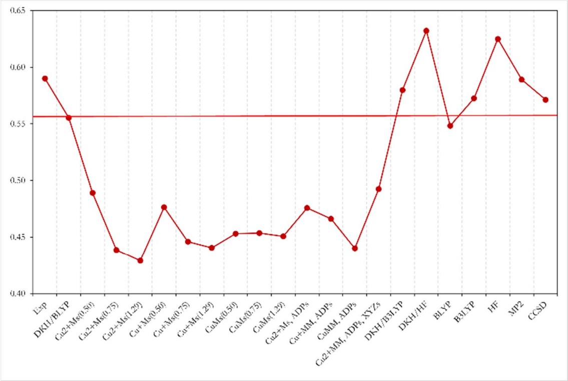

d) H5[c]

Figure S5 Graphical representation of AIM charges for all HC and QM methods; average values of: [a] acetate oxygens, [b] acetate carbons, [c] water hydrogens
